# Comparison of physical interventions, behavioral interventions, natural health products, and pharmacologics to manage hot flashes in patients with breast or prostate cancer: protocol for a systematic review incorporating network meta-analyses

**DOI:** 10.1186/s13643-015-0099-y

**Published:** 2015-08-27

**Authors:** Brian Hutton, Fatemeh Yazdi, Louise Bordeleau, Scott Morgan, Chris Cameron, Salmaan Kanji, Dean Fergusson, Andrea Tricco, Sharon Straus, Becky Skidmore, Mona Hersi, Misty Pratt, Sasha Mazzarello, Melissa Brouwers, David Moher, Mark Clemons

**Affiliations:** Ottawa Hospital Research Institute, Ottawa, Canada; University of Ottawa School of Epidemiology, Public Health and Preventive Medicine, Ottawa, Canada; Department of Oncology, McMaster University, Hamilton, Ontario Canada; Department of Medicine, Division of Medical Oncology, The Ottawa Hospital, Ottawa, Canada; Division of Radiation Oncology, Department of Radiology, University of Ottawa, Ottawa, Canada; Cornerstone Research Group, Toronto, Canada; Li Ka Shing Knowledge Institute, St Mike’s Hospital, Toronto, Canada

**Keywords:** Breast cancer, Prostate cancer, Hot flash, Systematic review, Network meta-analysis

## Abstract

**Background:**

Breast and prostate cancers are the most commonly diagnosed non-dermatologic malignancies in Canada. Agents including endocrine therapies (e.g., aromatase inhibitors, gonadotrophin-releasing hormone analogs, anti-androgens, tamoxifen) and chemotherapy have improved survival for both conditions. As endocrine manipulation is a mainstay of treatment, it is not surprising that hot flashes are a common and troublesome adverse effect. Hot flashes can cause chills, night sweats, anxiety, and insomnia, lessening patients’ quality of life. These symptoms impact treatment adherence, worsening prognosis. While short-term estrogen replacement therapy is frequently used to manage hot flashes in healthy menopausal women, its use is contraindicated in breast cancer. Similarly, testosterone replacement therapy is contraindicated in prostate cancer. It is therefore not surprising that non-hormonal pharmacological treatments (anti-depressants, anti-epilectics, anti-hypertensives), physical/behavioral treatments (e.g., acupuncture, yoga/exercise, relaxation techniques, cognitive behavioral therapy), and natural health products (e.g., black cohosh, flax, vitamin E, ginseng) have been studied for control of hot flashes. There is a need to identify which interventions minimize the frequency and severity of hot flashes and their impact on quality of life. This systematic review and network meta-analysis of randomized studies will synthesize available evidence addressing this knowledge gap.

**Methods/design:**

An electronic search of Medline, Embase, AMED, PsycINFO, and the Cochrane Register of Controlled Trials has been designed by an information specialist and peer reviewed by a second information specialist. Study selection and data collection will be performed by two reviewers independently. Risk of bias assessments will be completed using the Cochrane Risk of Bias Scale. Outcomes of interest will include validated measures of hot flash severity, hot flash frequency, quality of life, and harms. Bayesian network meta-analyses will be performed where judged appropriate based on review of clinical and methodologic features of included studies.

**Discussion:**

Our review will include a broad range of interventions that patients with breast and prostate cancer have attempted to use to manage hot flashes. Our work will establish the extent of evidence underlying these interventions and will employ an inclusive approach to analysis to inform comparisons between them. Our findings will be shared with Cancer Care Ontario for consideration in the development of guidance related to supportive care in these patients.

**Systematic review registration:**

PROSPERO: CRD42015024286

**Electronic supplementary material:**

The online version of this article (doi:10.1186/s13643-015-0099-y) contains supplementary material, which is available to authorized users.

## Background

Breast and prostate cancers are the most commonly diagnosed non-dermatologic malignancies in Canada, with over 23,000 women and 23,000 men diagnosed annually [1, 2]. Agents including endocrine therapies (e.g., aromatase inhibitors, gonadotrophin-releasing hormone analogs, anti-androgens, and tamoxifen), endocrine manipulations (e.g., surgical ovarian removal), and chemotherapy (with resulting premature ovarian failure) have improved survival for both conditions. As endocrine manipulation is the mainstay of treatment for both diseases, it is not surprising that hot flashes are a troublesome side effect in two of every three patients receiving endocrine therapy [3]. Hot flashes are a vasomotor symptom which lessens patients’ quality of life given that they cause chills, night sweats, and irregular heartbeat and can cause compromised sleep and insomnia [4–6]. They can be a challenge for years. These symptoms impact adherence to treatment, with non-adherence rates as high as 55 %, and can therefore worsen prognosis [4]. While estrogen replacement therapy (ERT) is often used for hot flashes in healthy menopausal women, its use is contraindicated in breast cancer [7]. Similarly, testosterone replacement therapy (TRT) is contraindicated in prostate cancer [8–10]. Hence, non-hormonal pharmacological treatments (e.g., anti-depressants, anti-epilectics, anti-hypertensives), physical/behavioral treatments (e.g., yoga/exercise, relaxation techniques, cognitive behavioral therapy), acupuncture, and natural health products (e.g., black cohosh, flax, vitamin E, ginseng) have been studied for control of hot flashes [11–13]. Establishing which non-hormonal treatments can minimize the frequency and severity of hot flashes and their impact on quality of life is vital for prostate and breast cancer survivors. We will perform a systematic review with network meta-analyses to address the following question: In breast cancer and prostate cancer survivors, what are the relative benefits of non-hormonal therapies on (1) frequency and severity of hot flashes? (2) quality of life? (3) quality of life related to depression and sleep quality? While past reviews have considered some of these treatments in isolation, there is a need for network meta-analyses [14–16] to compare many alternatives in a unified analysis.

## Methods/design

This protocol has been registered within the PROSPERO database for systematic reviews (CRD42015024286).

### Data sources and search for studies

The search strategy has been developed and tested through an iterative process by an experienced medical information specialist in consultation with the review team. Using the OVID platform, we will search Ovid MEDLINE®, Ovid MEDLINE® In-Process & Other Non-Indexed Citations, Embase, AMED, and PsycINFO. We will also search the CENTRAL database using the Cochrane Library on Wiley. Strategies will utilize a combination of controlled vocabulary (e.g., “Breast Neoplasms,” “Prostatic Neoplasms,” “Hot Flashes”) and keywords (e.g., breast cancer, prostate cancer, vasomotor symptoms). Vocabulary and syntax will be adjusted across databases. No language or date restrictions will be applied. We will use a validated randomized controlled trial filter and will remove animal-only and opinion-pieces from the results. The core strategy will be reviewed prior to execution by another senior information specialist using the Peer Review for Electronic Search Strategies checklist [17]. We will search the World Health Organization’s ICTRP Search Portal and www.ClinicalTrials.gov for randomized controlled trials and will also hand-search the bibliographies of pertinent references. Specific details regarding the strategies appear in Additional file 1.

### Study eligibility criteria

Studies fulfilling the following PICOS (Population-Intervention-Comparators-Outcomes-Study design) criteria will be selected for inclusion in this review.

#### Population

Studies involving patients diagnosed with breast cancer or prostate cancer and who are experiencing hot flashes will be sought. No restrictions on age or cancer stage will be used.

#### Interventions and comparators

**Fig. 1:**
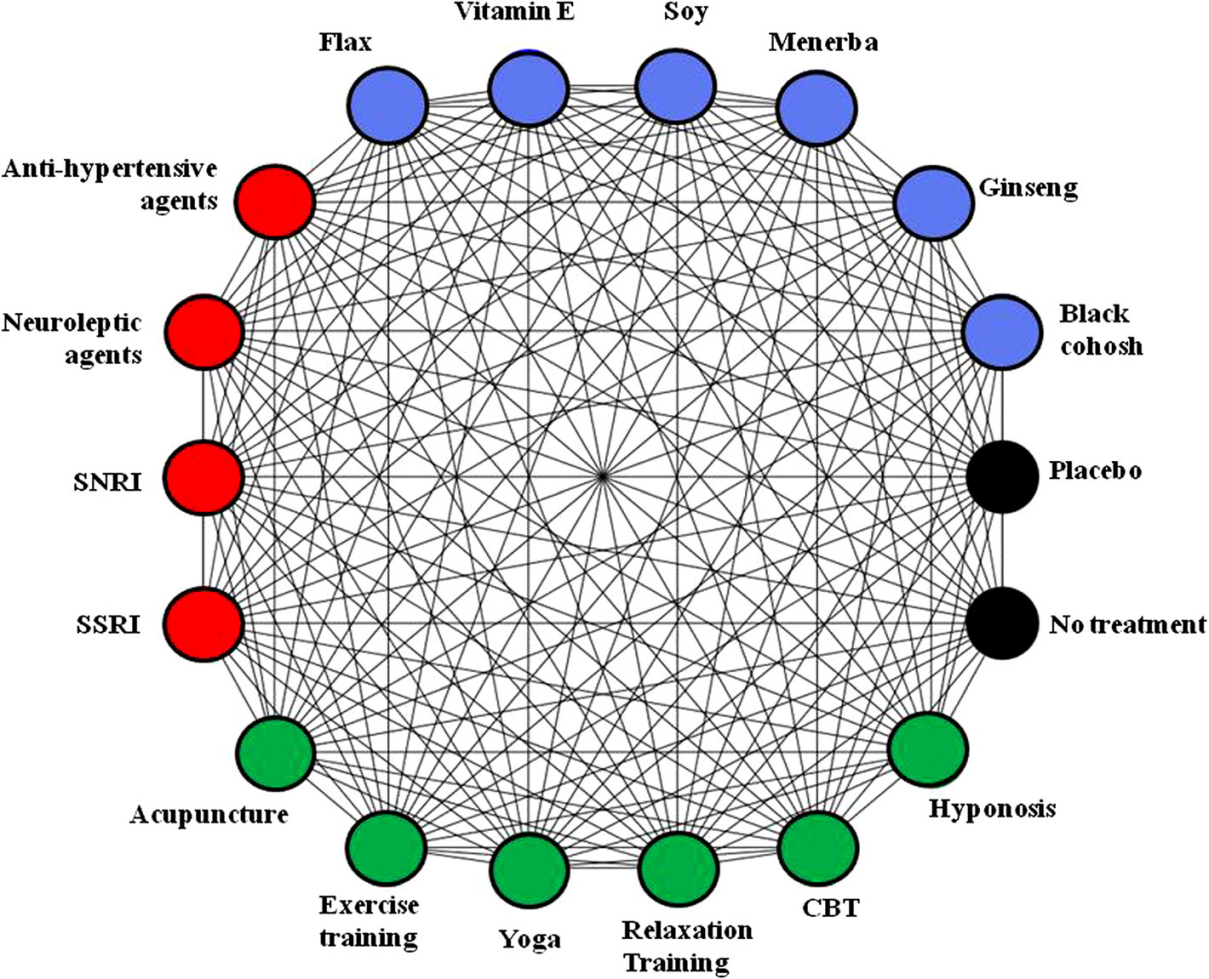
Interventions eligible for network meta-analyses in the review. The colors reflect treatments of similar groupings: red = pharmacologic interventions, blue = natural health products, and green = physical activities and behavioral therapies. Lines reflect where comparisons may exist between treatments. Which comparisons have been studied will be established by study selection. Availability of outcomes can also impact network structure. Network refinement to reflect dose ranges and categories of treatments will be established with the guidance of clinical experts. *SSRI* selective serotonin reuptake inhibitor, *SNRI* selective norepinephrine reuptake inhibitor, *CBT* cognitive behavioral therapy

Studies evaluating the effects of non-hormonal pharmacologic, behavioral/physical, and natural health product interventions will be considered. Eligible pharmacologic interventions will include anti-depressants from the selective serotonin reuptake inhibitors (SSRI) class (e.g., sertraline, escitalopram, citalopram, and so forth) and selective norepinephrine reuptake inhibitor (SNRI) class (e.g., venlafaxine, duloxetine, and so forth), as well as certain neuroleptic agents (gabapentin, clonidine) and anti-hypertensive medications (e.g., alpha blockers, beta blockers). All doses and formulations will be eligible. Structured physical/behavioral interventions of interest will include acupuncture, yoga, exercise programs, hypnosis, relaxation techniques, and cognitive behavioral therapy. Nutritional healthcare products of interest will include vitamin E, flax, ginseng, black cohosh, isoflavones, menerba, and soy. All doses and formulations will be eligible. Lastly, placebo and no treatment will be eligible given their high likelihood of being links to indirect evidence in the treatment network. We will treat them as separate interventions in our analyses. After discussion with the clinical and statistical experts on the team, the nodes will be finalized to maximize clinical relevance. For example, additional nodes might be added to account for combinations of the above therapies. Refinements in terms of dosage stratifications, representation of differences in implementation of physical/behavorial interventions or other factors for all of the above interventions will be made in collaboration with our team of clinical experts to maximize clinical relevance once data collection is completed. Figure 1 presents a preliminary schematic diagram of the treatments to be studied.

#### Outcomes

The primary outcomes of interest will be changes in the intensity and frequency of hot flashes, with or without night sweats. Changes in quality of life are also of interest in terms of both symptom-specific and generic measures. More specifically, changes of hot flash intensity are typically measured using scales such as the Hot Flash Daily Related Interference Scale [18], the Greene Climacteric Score [19], and the Modified Kupperman Index, [20] amongst others; we will collect intensity outcomes reported using any validated measure. The reporting of hot flash frequency varies amongst trials and may be reported as the percentage change in the frequency of hot flashes from baseline, the mean number of hot flashes per day, or the percentage of patients remaining free of hot flashes during the study; we will collect information for each of these measures. Regarding symptom-specific and generic quality of life measures, a range of scales have been used in trials of hot flash interventions, including symptom-specific measures for depression and sleep quality (e.g., the Center for Epidemiologic Studies Depression Scale (CES-D) [21], Insomnia Severity Index [22]) as well as generic measures (e.g., EuroQOL Linear Rating Scale [23]); we will collect data from validated symptom-specific and generic quality of life (QoL) scales for use in separate analyses. Table 1 presents an overview of outcomes of interest related to these clinical manifestations identified by our research team that may be encountered during the review. Secondary outcomes to be collected will include measures related to adherence to cancer therapies and harms associated with each treatment (e.g., drug toxicities, discontinuation rates, suicidal behaviors).

**Table 1:** Validated generic and symptom-specific scales for the review

Outcome	Related validated scales for data collection
Hot flash severity	Scores with subscale for hot flashes severity
	The Greene Climacteric Score
	Hot Flash Daily Related Interference Scale (HFDRIS)
	Menopause Rating Scale (MRS)
	The Mayo Clinic Hot flash Index
Generic quality of life	EURO-QOL Linear Rating Scale
	Hot Flash Daily Related Interference Scale (HFDRIS)
	Menopause Specific Quality of Life Questionnaire (MENQOL) (also the modified version)
	SF-12 Health Survey
	SF-36 Health Survey
	The Psychological General Well-Being Index (PGWBI)
	The Greene Climacteric Score
	Menopause Rating Scale (MRS)
Depression symptoms	Scores with subscale for depression
	Menopause Rating Scale (MRS)
	The profile of mood state (POMS)
	Center for Epidemiologic Studies Depression Scale (CES-D)
	Hamilton Depression Rating Scale
	Montgomery-Åsberg Depression Rating Scale
	The Primary Care Evaluation of Mental Disorders (PRIME-MD)
	Beck depression inventory-II (BDI-II)
	Patient Health Questionnaire (PHQ) (9-PHQ, and 2-PHQ self reported versions)
	Raskin Depression Rating Scale
Sleep quality symptoms	Scores with subscale for insomnia
	Menopause Rating Scale (MRS)
	Insomnia Severity Index (ISI)
	Pittsburgh Sleep Quality Index
	Epworth Sleepiness Scale
	Basic Nordic Sleep Questionnaire (BNSQ)
	Stanford Sleepiness Scale
	Brief Insomnia Questionnaire

Validated scales of relevance related to hot flash severity, frequency, and other outcomes of interested identified by participating experts as possibly arising in studies relevant to the review are shown. If additional validated scales arise during data collection, these will also be included

#### Study design

We will include randomized controlled trials, including those of open-label design. Both parallel group and crossover trials will be eligible. For crossover trials, we will make use of data from the first study period due to the potential for carryover effects. We will exclude quasi-randomized and non-randomized studies.

### Screening and data extraction

Four reviewers will work in pairs to review abstracts (Stage 1 screen) and full text reports (Stage 2 screen) from search results independently and in duplicate against the eligibility criteria using Distiller SR software (Ottawa, Canada) to identify relevant articles. Both stages of screening will begin with a calibration exercise of approximately 50 abstracts and 10 full text reports to ensure consistent application of eligibility criteria. Study selection will be documented using a preferred reporting items for systematic reviews and meta-analysis (PRISMA) flow diagram [24]. Once all studies are identified, data extraction will be performed using a standardized extraction form which will be piloted by the reviewers on a small number of studies. We will collect data related to key items including patient demographics (age, sex, cancer stage, duration of hot flashes before randomization, concurrent therapies, baseline hot flash frequency and severity, etc.), interventions (doses, frequency of administration, setting, experience of therapist (when relevant), etc.), and outcomes as described above. We will use the Cochrane Risk of Bias Tool [25] for randomized controlled trials (RCTs) to establish the risk of bias of each included study; a summary of findings from these assessments will be provided, and they will also be used to consider sensitivity analyses.

### Approach to evidence synthesis

Meta-analyses will be conducted in breast cancer and prostate cancer patients separately and collectively. This approach will enable inspection of the comparability of effects of interventions in both populations while also allowing for a more inclusive and well powered analysis of the available data. We will summarize characteristics of included studies focusing on clinical (e.g., diagnosis, age, sex, number of and types of prior therapies, duration of disease, time since onset of hot flashes) and methodologic (e.g., risk of bias, design) homogeneity, and we will review the distribution of these treatment effect modifiers across studies and comparisons in the treatment network to assess the validity of the assumptions of homogeneity, similarity, and consistency [26]. Where there is homogeneity of important effect modifiers, we will perform network meta-analyses (NMAs) to compare interventions [14–16].

Prior to NMAs, we will perform pairwise meta-analyses for each comparison in the treatment networks where studies are available to explore for heterogeneity based on the *I*^2^ statistic [27]. Fixed and random effects Bayesian NMAs will be performed using a common heterogeneity parameter as per established methods [15, 28]. Model fit will be assessed by comparing the model’s residual deviance with the number of unconstrained data points [28]. Selection between models will be based on deviance information criteria (DIC), with a difference of five points suggesting an important difference. Comparison of hot flash frequency is commonly expressed as average number per day and percentage change of frequency from baseline, and both will be analyzed using a model for mean differences (MD). We will consider a model for standardized mean differences (SMD) when assessing hot flash severity, changes in general QoL, and symptom-specific QoL measures, as it will be of interest to explore benefits across related scales to maximize available data. All findings will be presented with anchor-based (or if needed, distribution-derived) minimally important differences to provide insights on how estimated benefits compare to what may be clinically relevant for patients. For outcomes analyzed using an SMD summary measure, we will use established methods [29, 30] to present results in minimal important difference (MID) units such that findings are more interpretable for knowledge users. All pairwise comparisons between interventions will be expressed as MDs or SMDs with corresponding 95 % credible intervals. If the structure of treatment networks is judged to be sound (i.e., if there does not exist a large number of comparisons in the network with no available study data and there does not exist a high number of comparisons informed by single studies), we will supplement reporting of pairwise comparisons with Surface Under the Cumulative Ranking (SUCRA) curve values and median treatment rankings (with corresponding 95 % credible intervals). The assumption of consistency of direct and indirect data will be assessed by fitting inconsistency models as described elsewhere [31], which will include comparison of DIC from these models with DIC from the corresponding consistency models alluded to above; scatterplots of residuals from both models will also be inspected to identify potential studies contributing to inconsistency. All NMAs will be performed using Winbugs software version 1.4.3 (MRC Biostatistics). Model convergence will be assessed using established methods including Gelman Rubin diagnostics and inspection of Monte Carlo errors [28]. The short nature of trials in this area is likely to be associated with a lack of reporting of adherence data for cancer therapies. If this data is available, we will employ similar modeling approaches for binary data to compare the numbers of patients maintaining adherence to primary therapies. As the types of harms are likely to vary due to the different types of interventions being compared, meta-analyses for specific harms may not be feasible. If this is true, we will present descriptions of the harms for each intervention.

Regarding sources of heterogeneity, we will explore subgroup analyses and meta-regression analyses to address the impact of covariates on our findings to establish their robustness [32, 33]. Based on clinical expertise and the existing literature, the following group-level factors will be explored: percentage of male participants, average patient age, percentage receiving concurrent hormone therapy, percentage with history of chemotherapy, average time since first hot flash, and average time since cancer diagnosis; others may be added if factors seen as important by our clinical experts emerge during data collection and review of study characteristics. Regarding network structure, we will explore sensitivity analyses using alternative geometries [34] which may include refining how interventions are reflected in the treatment network. For example, if experts on the team consider doses of a drug to be importantly different across studies, these treatments will be considered distinct from each other in analyses to maximize clinical relevance. Conversely, analyses lumping treatments into one group (e.g., all SSRIs together) may also be considered. Findings from all analyses will be reported. For connections in the treatment network consisting of ten studies or more, we will review funnel plots to explore for the potential presence of publication bias.

### Reporting of review findings

Reporting of findings from this systematic review will adhere to recommendations from the PRISMA extension statement for network meta-analysis [35]. Recommended graphical approaches including forest plots, league tables, and rank-o-grams will be used [36]. We will prepare a summary of the geometries of the networks in breast and prostate cancer as seen for other conditions [37–39] to provide insight on future research needs for trialists.

## Discussion

Many different interventions including natural health products, drugs, physical activities, and behavioral therapies have been studied in hopes of identifying novel approaches to reducing the impact that vasomotor symptoms such as hot flashes have on patients’ quality of life. There is a need to compare hot flash treatments in cancer patients to inform guidance, improve care, and share information with patients. Given that many non-hormonal treatments exist, a network meta-analysis [14–16] is needed to establish relative benefits for patients. We will employ dedicated methods to enhance interpretability for patients and decision-makers [40]. We will share our findings with Cancer Care Ontario’s Program in Evidence Based Care, which will incorporate them into future clinical guidance in their Supportive Care program.

Currently, guidance related to interventions for management of hot flashes in breast and prostate cancers is limited and inconsistent. While reviews comparing some of the above interventions have been conducted, several are in need of updating, and thus far, no network meta-analyses to compare the benefits of all existing interventions have been performed to address this knowledge gap. By conducting the proposed review, we hope to improve clinicians’ and patients’ awareness regarding the relative benefits of different interventions to manage hot flashes. We intend to address this important knowledge gap in the management of supportive care for patients with breast and prostate cancers by conducting this study to (1) generate rigorous scientific publications describing our findings in open access journals to target clinicians, nurses, and patients alike; (2) bring our findings to the table for use amongst clinical guidance developers at Cancer Care Ontario for development of future guidance; and (3) generate targeted lay summaries of our findings for clinicians and patients to make our results accessible. We believe our planned deliverables are capable of impacting future management of breast and prostate cancer survivors who experience hot flashes.

## Additional file

Additional file 1:
**Draft search.** Description: Contains the search strategy to be used for the planned systematic review. (DOCX 17 kb)

## Abbreviations

DIC: Deviance information criteria
ERT: Estrogen replacement therapy
MD: Mean difference
MID: Minimal important difference
NMA: Network meta-analysis
PRISMA: Preferred reporting items for systematic reviews and meta-analysis
QoL: Quality of life
RCT: Randomized controlled trial
SMD: Standardized mean difference
SNRI: Selective norepinephrine reuptake inhibitor
SSRI: Selective serotonin reuptake inhibitor
SUCRA: Surface under the cumulative ranking
TRT: Testosterone replacement therapy
