# Supplementary material for: Comparison of physical interventions, behavioral interventions, natural health products, and pharmacologics to manage hot flashes in patients with breast or prostate cancer: protocol for a systematic review incorporating network meta-analyses
Source: Syst Rev. 2015 Aug 27;4:114. doi: 10.1186/s13643-015-0099-y (PMC4549873; doi:10.1186/s13643-015-0099-y)
Supplement: Additional file 1: — Draft search. Description: Contains the search strategy to be used for the planned systematic review. (DOCX 17 kb) [file 13643_2015_99_MOESM1_ESM.docx]

**Additional file 1: Search Strategy**

Database: AMED (Allied and Complementary Medicine) <1985 to June 2015>, Embase Classic+Embase <1947 to 2015 June 26>, Ovid MEDLINE(R) In-Process & Other Non-Indexed Citations and Ovid MEDLINE(R) <1946 to Present>, PsycINFO <1806 to June Week 4 2015>

Search Strategy:

--------------------------------------------------------------------------------

1 exp Breast Neoplasms/

2 ((breast* or mamma or mammar*) adj3 (cancer* or carcinoid* or carcinoma* or carcinogen* or adenocarcinoma* or adeno-carcinoma* or malignan* or neoplasia* or neoplasm* or sarcoma* or tumour* or tumor*)).tw,kw.

3 exp Prostatic Neoplasms/

4 ((prostate or prostatic) adj3 (cancer* or carcinoid* or carcinoma* or carcinogen* or adenocarcinoma* or adeno-carcinoma* or malignan* or neoplasia* or neoplasm* or sarcoma* or tumour* or tumor*)).tw,kw.

5 or/1-4

6 Hot Flashes/

7 (hot flash* or hot flush*).tw,kw.

8 night sweat*.tw,kw.

9 ((vasomotor or vaso-motor) adj5 (disorder* or disturbance* or instabilit* or symptom*)).tw,kw.

10 ((climacteri* or menopaus* or premenopaus* or pre-menopaus* or postmenopaus* or post-menopaus*) adj5 (disorder* or disturbance* or instabilit* or symptom*)).tw,kw.

11 or/6-10

12 5 and 11

13 (controlled clinical trial or randomized controlled trial).pt.

14 clinical trials as topic.sh.

15 (randomi#ed or randomly or RCT$1 or placebo*).tw.

16 ((singl* or doubl* or trebl* or tripl*) adj (mask* or blind* or dumm*)).tw.

17 trial.ti.

18 or/13-17

19 12 and 18

20 exp Animals/ not (exp Animals/ and Humans/)

21 19 not 20

22 (comment or editorial or interview or news).pt.

23 (letter not (letter and randomized controlled trial)).pt.

24 21 not (22 or 23)

25 24 use prmz [MEDLINE]

26 exp breast tumor/

27 ((breast* or mamma or mammar*) adj3 (cancer* or carcinoid* or carcinoma* or carcinogen* or adenocarcinoma* or adeno-carcinoma* or malignan* or neoplasia* or neoplasm* or sarcoma* or tumour* or tumor*)).tw,kw.

28 exp prostate tumor/

29 ((prostate or prostatic) adj3 (cancer* or carcinoid* or carcinoma* or carcinogen* or adenocarcinoma* or adeno-carcinoma* or malignan* or neoplasia* or neoplasm* or sarcoma* or tumour* or tumor*)).tw,kw.

30 or/26-29

31 hot flush/

32 (hot flash* or hot flush*).tw,kw.

33 night sweat*.tw,kw.

34 vasomotor disorder/

35 ((vasomotor or vaso-motor) adj5 (disorder* or disturb* or instabilit* or symptom*)).tw,kw.

36 ((climacteri* or menopaus* or premenopaus* or pre-menopaus* or postmenopaus* or post-menopaus*) adj5 (disorder* or disturb* or instabilit* or symptom*)).tw,kw.

37 or/31-36

38 30 and 37

39 randomized controlled trial/ or controlled clinical trial/

40 exp "clinical trial (topic)"/

41 (randomi#ed or randomly or RCT$1 or placebo*).tw.

42 ((singl* or doubl* or trebl* or tripl*) adj (mask* or blind* or dumm*)).tw.

43 trial.ti.

44 or/39-43

45 38 and 44

46 exp animal experimentation/ or exp models animal/ or exp animal experiment/ or nonhuman/ or exp vertebrate/

47 exp humans/ or exp human experimentation/ or exp human experiment/

48 46 not 47

49 45 not 48

50 editorial.pt.

51 letter.pt. not (letter.pt. and randomized controlled trial/)

52 49 not (50 or 51)

53 52 use emczd [EMBASE]

54 exp Breast Neoplasms/

55 ((breast* or mamma or mammar*) adj3 (cancer* or carcinoid* or carcinoma* or carcinogen* or adenocarcinoma* or adeno-carcinoma* or malignan* or neoplasia* or neoplasm* or sarcoma* or tumour* or tumor*)).tw.

56 exp Prostatic Neoplasms/

57 ((prostate or prostatic) adj3 (cancer* or carcinoid* or carcinoma* or carcinogen* or adenocarcinoma* or adeno-carcinoma* or malignan* or neoplasia* or neoplasm* or sarcoma* or tumour* or tumor*)).tw.

58 or/54-57

59 Hot Flashes/

60 (hot flash* or hot flush*).tw,kw.

61 night sweat*.tw.

62 ((vasomotor or vaso-motor) adj5 (disorder* or disturbance* or instabilit* or symptom*)).tw.

63 ((climacteri* or menopaus* or premenopaus* or pre-menopaus* or postmenopaus* or post-menopaus*) adj5 (disorder* or disturbance* or instabilit* or symptom*)).tw.

64 or/59-63

65 58 and 64

66 (controlled clinical trial or randomized controlled trial).pt.

67 exp Clinical Trials/

68 (randomi#ed or randomly or RCT$1 or placebo*).tw.

69 ((singl* or doubl* or trebl* or tripl*) adj (mask* or blind* or dumm*)).tw.

70 trial.ti.

71 or/66-70

72 65 and 71

73 exp Animals/ not (exp Animals/ and Humans/)

74 72 not 73

75 (comment or editorial or interview or news).pt.

76 (letter not (letter and randomized controlled trial)).pt.

77 74 not (75 or 76)

78 77 use amed [AMED]

79 breast neoplasms/

80 ((breast* or mamma or mammar*) adj3 (cancer* or carcinoid* or carcinoma* or carcinogen* or adenocarcinoma* or adeno-carcinoma* or malignan* or neoplasia* or neoplasm* or sarcoma* or tumour* or tumor*)).tw.

81 Prostate/ and exp Neoplasms/

82 ((prostate or prostatic) adj3 (cancer* or carcinoid* or carcinoma* or carcinogen* or adenocarcinoma* or adeno-carcinoma* or malignan* or neoplasia* or neoplasm* or sarcoma* or tumour* or tumor*)).tw.

83 or/79-82

84 (hot flash* or hot flush*).tw,kw.

85 night sweat*.tw.

86 ((vasomotor or vaso-motor) adj5 (disorder* or disturb* or instabilit* or symptom*)).tw,kw.

87 ((climacteri* or menopaus* or premenopaus* or pre-menopaus* or postmenopaus* or post-menopaus*) adj5 (disorder* or disturb* or instabilit* or symptom*)).tw,kw.

88 or/84-87

89 83 and 88

90 clinical trials/

91 (randomi#ed or randomly or RCT$1 or placebo*).tw.

92 ((singl* or doubl* or trebl* or tripl*) adj (mask* or blind* or dumm*)).tw.

93 trial.ti.

94 or/90-93

95 89 and 94

96 exp Animals/ not (exp Animals/ and Humans/)

97 95 not 96

98 97 use prmz

99 97 use emczd

100 97 use amed

101 97 not (98 or 99 or 100) [PSYCINFO]

102 25 or 53 or 78 or 101

103 remove duplicates from 102 [UNIQUE RECORDS]

104 103 use prmz [MEDLINE UNIQUE RECORDS]

105 103 use emczd [EMBASE UNIQUE RECORDS]

106 103 use amed [AMED UNIQUE RECORDS]

107 103 not (104 or 105 or 106) [PSYCINFO UNIQUE RECORDS]
